# Effects of diaphragm electrical stimulation in treating respiratory dysfunction on mechanical ventilation after intracerebral hemorrhage: A single-center retrospective study

**DOI:** 10.1097/MD.0000000000036767

**Published:** 2024-01-05

**Authors:** Yan Wu, Suqin Wang, Jing Zhang, Yan Wang, Jiaojiao Zhong, Yuhai Wang

**Affiliations:** a Department of Neurosurgery, The 904^th^ Hospital of Joint Logistic Support Force (The 101^st^ Hospital of PLA), Wuxi, China; b Department of Nursing, The 904^th^ Hospital of Joint Logistic Support Force (The 101^st^ Hospital of PLA), Wuxi, China.

**Keywords:** electrical stimulation, ICH, mechanical ventilation, respiratory dysfunction

## Abstract

Intracerebral hemorrhage (ICH) is a major cause of death and disability worldwide. The benefits of electrical stimulation in the treatment of respiratory dysfunction in patients on mechanical ventilation is unknown. Nevertheless, there is a dearth of evidence-based medical research concerning its clinical efficacy. From January 2019 to January 2023, every enrolled patients experienced respiratory dysfunction after ICH while being supported by mechanical ventilation. A total of 205 eligible patients were enrolled and then allocated into 2 groups: control group and observation group. 133 patients was selected and administered standard treatment as control group. Based on conventional treatment, other 72 patients were administered diaphragm electrical stimulation (DES) treatment. We examined information from current medical records, encompassing all initial data and predictive follow-up data, such as the weaning success rate, occurrence of ventilator-associated pneumonia (VAP), duration of stay in the intensive care unit (ICU) and hospital, expenses related to hospitalization, and mortality within 30 days. The baseline clinical data of the 2 groups did not exhibit any statistically significant disparities (all *P* > .05). The rate of successful weaning showed a significant increase in the DES group when compared to the control group (*P* = .025). In patients with respiratory dysfunction due to ICH, treatment with DES resulted in a significant reduction in the duration of invasive ventilation (9.8 ± 2.1 vs 11.2 ± 2.6, *P* < .01) and total ventilation time (9.8 ± 2.1 vs 11.2 ± 2.6, *P* < .01). It also led to a decrease in the length of stay in the ICU (15.67 ± 3.76 vs 17.53 ± 4.28, *P* = .002) and hospitalization cost (11500 vs 13600, *P* = .001). Additionally, DES treatment resulted in a lower incidence of VAP (73.61% vs 86.46%, *P* = .022) and improved 30-day mortality (*P* < .05), without any significant adverse effects. The findings of this research indicate that DESs have a positive impact on enhancing the rate of successful weaning and reducing the incidence of VAP. It decreases the duration of invasive ventilation and total ventilation time while also improving the mortality rate within 30 days. This therapy could offer a fresh alternative for respiratory impairment in patients undergoing mechanical ventilation.

## 1. Introduction

With age, the occurrence of spontaneous intracerebral hemorrhage (ICH), the most fatal type of stroke, rises, making up approximately 15 to 20% of all strokes.^[1–4]^ Severe ICH caused by a substantial intracranial hematoma is linked to significant morbidity and mortality, as it can cause initial brain damage by damaging brain tissue and increasing intracranial pressure (ICP) due to the large hematoma.^[5,6]^ The reported mortality rate for ICH within 30 days is between 30% and 55%, with approximately half of the fatalities occurring in the acute phase, particularly within the initial 48 hours following the start of symptoms.^[7,8]^ Chen also reported that more than 35% of ICH patients need long-term mechanical ventilation treatment.^[9]^ The percentage may be even higher in patients with brainstem hemorrhages. Long-term use of the ventilator may lead to ventilator dependence and difficulty in weaning. Currently, treatment of ventilator dependence is challenging, and a viable treatment strategy is lacking.

Respiratory dysfunction after ICH is an important cause of ventilator dependence, and the main manifestation is respiratory muscle weakness, especially the obvious decline in diaphragm function, decreased lung capacity, insufficient chest expansion, reduced chest lung compliance, and decreased cough and expectoration ability. Subsequently, the individual may experience repeated respiratory tract infections and atrial dilatation, leading to respiratory failure or potentially resulting in fatality.^[10,11]^ Breathing involves the diaphragm and abdominal muscles, both of which play a crucial role. The diaphragm serves as the primary respiratory muscle. Diaphragm electrical stimulation (DES) can improve lung ventilation by making the diaphragm contract regularly during rehabilitation. According to Bao,^[12]^ DESs were predominantly utilized in clinical settings to enhance muscle power and flexibility, alleviate swelling, prevent muscle wasting, alleviate discomfort, and ultimately facilitate tissue recovery. A recent meta systematic review also summarized the value of DES, which can also decrease the duration of invasive mechanical ventilation, while the quality of the body of evidence is low to very low, need more larger number sample randomized controlled trials to confirmed.^[13]^ It is, however, difficult to determine if DES has a therapeutic effect on patients suffering from ICH-related respiratory function in patients on mechanical ventilation.

However, because of the unique traits of people with ICH and the lack of extensive research with large sample sizes, the efficacy of DES remains unclear. Hence, the present study sought to investigate the hypothesis that implementing DES could improve the results of respiratory function in patients on mechanical ventilation.

## 2. Methods

### 2.1. Study design

A retrospective study was carried out at Wuxi Taihu Hospital in Jiangsu, China, from January 2019 to January 2023. Throughout this timeframe, a screening process was carried out on a total of 250 individuals who were receiving mechanical ventilation for respiratory function following ICH. Among them, 205 participants were initially included in the research. The methodology employed in the current research (2019-YXLL-37) was approved by the Wuxi Taihu Hospital Clinical Research Ethics Committees and adhered to the principles outlined in the Declaration of Helsinki. Participants were assigned to either the observation group or the control group. During the same time frame, a control group consisting of 133 patients was selected and administered standard treatment. Conventional treatment was used to administer DES treatment to the remaining 72 patients in the observation group.

### 2.2. Patients enrolled in the study and sample selection procedures

The current study included individuals in the intensive care unit (ICU). The research team approached the individuals, evaluated their suitability, obtained consent after providing information, and registered them. The eligibility requirements were as follows: individuals aged 18 to 75; and confirmed diagnosis of respiratory dysfunction in patients undergoing mechanical ventilation after ICH. The exclusion criteria consisted of: patients who were not anticipated to improve upon admission; individuals under 18 or over 75 years old; pregnant women; absence of requirement for a ventilator; patients with existing neuromuscular injuries; and patients currently undergoing palliative care and those with a life expectancy of <7 days.

### 2.3. Technique of DES

In this article, we will briefly review the procedure technique of diaphragm pacing that has been described previously.^[14]^ In the study group, patients were administered the DES intervention for 30 minutes per day using a portable device called ResPower Respiratory Neuromuscular Stimulator, manufactured in Yaguo, China. To measure the movement of the pectoralis major muscle, rectus abdominis, and bilateral quadriceps muscles, a negative electrode was positioned. The positive electrodes were positioned at a distance from the initial electrode, close to the targeted muscle for stimulation. Each muscle is equipped with 2 electrodes. The given parameters included a frequency of 50 Hz, a pulse duration of 300 ms, a rise time of 1 second, a stimulus time of 3 seconds, a decay time of 1 second, and a relaxation time of 10 seconds. The intensity of electric stimulation was gradually increased until observable muscle contractions were experienced. It was recommended that patients tolerate as much electrical stimulation as possible.

### 2.4. Outcome assessment

A committee, which was independent and responsible for diagnosis and assessment, thoroughly examined all clinical and imaging data along with the treatment provided. Before the start of the current study, 2 researchers and 1 nurse, who had undergone training, formed the committee and had no role in providing clinical care to patients. We examined information from current medical records, encompassing all initial data and predictive follow-up data. The primary aim of this research was to determine the success rate of weaning. Furthermore, we assessed the occurrence of ventilator-associated pneumonia (VAP). The secondary objective was to assess the 6-month Glasgow Outcome Scale (GOS) score and the mortality rate within 30 days. The results were classified by GOS into 5 levels, with grade 5 representing a favorable recovery, grade 4 indicating a slight impairment, grade 3 indicating a significant disability, grade 2 indicating a persistent vegetative state, and grade 1 indicating death.^[15,16]^

### 2.5. Assessment of safety and potential issues

We observed the length of time patients stayed in the ICU, and the main adverse effects of DES included visible signs of tissue or neurovascular injury. After the verification and recording of all complications by 2 physicians and medical personnel. The assessment involved palpating the distal radial pulses, visually examining for local swelling, redness, and skin sores, and checking for sensitivity through palpation.

### 2.6. Hospital stays and the expenses associated with hospitalization

Individuals experiencing respiratory impairment following ICH typically endure an extended duration of hospitalization, resulting in exorbitant healthcare expenses. Significant decreases in hospital stays and hospitalization costs would be observed if there is an improvement in complications and prognosis, as well as early weaning. The objective of this research was to examine the difference in the overall duration of hospitalization, length of stay in the ICU, and healthcare costs among individuals belonging to the 2 categories.

### 2.7. Statistical analysis

Continuous variables were expressed using means and standard deviations. The statistical analyses were performed using SPSS software (version 20.0) and GraphPad Prism 6.0 (GraphPad Software, Inc., www.graghpad.com/prism). The data committee verified all of the data. The evaluation of quantitative data was conducted through independent-sample t-tests. To compare qualitative data, either the chi-square test or Fisher exact t-test was employed. Statistical significance was established when the *P* value was below .05.

## 3. Results

### 3.1. Clinical data and baseline assessment

From January 2019 to January 2023, an extensive evaluation was carried out on 250 people experiencing respiratory impairment following ICH. Out of the total, 205 individuals were initially selected and divided into 2 groups: 1 receiving DES treatment (n = 72) and the other receiving a control treatment (n = 133). Furthermore, no significant differences were observed in the baseline data between the 2 groups (Table 1). The evaluation of all patients included in the current study was ultimately completed. The ultimate follow-up for the most recent patient will occur on September 30, 2023.

**Table 1:** Comparison of baseline data.

	DES Group (n = 72)	Control Group (n = 133)	*P*
Age (Y, mean ± SD)	53.4 ± 11.7	54.5 ± 12.5	.539
Gender, no. (%)			.766
Male	40 (55.56%)	71 (53.38%)	
Female	32 (44.44%)	62 (46.62%)	
BMI (KG/cm^2^, mean ± SD)	21.8 ± 1.9	22.1 ± 2.1	.314
GCS at admission			.820
3–5	29 (40.28%)	57 (52.86%)	
6–8	34 (47.22%)	63 (47.37%)	
9–15	9 (12.50%)	13 (9.77%)	
Mydriasis			.725
No	37 (51.39%)	70 (52.63%)	
Single	20 (27.78%)	40 (30.08%)	
Bilateral	15 (20.83%)	23 (17.29%)	
ICH location			.908
Brain stem	22 (30.56%)	44 (33.08%)	.712
Basal ganglia	31 (43.06%)	57 (42.86%)	.978
cerebellum	10 (13.89%)	18 (13.53%)	.944
others	9 (12.50%)	14 (10.53%)	.669
Surgery, no. (%)	42 (58.33%)	79 (59.40%)	.882
Smoking History, no. (%)	29 (40.28%)	60 (45.11%)	.404
Living environment, no. (%)			.869
Town	43 (59.72%)	81 (60.90%)	
Countryside	29 (40.28%)	52 (39.10%)	
Past medical history, no. (%)			
Hypertension	61 (84.72%)	109 (81.95%)	.615
Hyperlipidemia	35 (48.61%)	62 (46.62%)	.785
Diabetes	27 (37.50%)	44 (33.08%)	.209
APACHE II Score	19.7 ± 6.1	20.1 ± 6.2	.658
MOF, no. (%)	33 (45.83%)	59 (44.36%)	.840

BMI = body mass index, CT = computerized tomography, Glasgow coma scale, ICH = intracerebral hemorrhage, SD = standard deviation, MOF = multi-organ failure.

### 3.2. The primary aim

Following a period of 6 months, the evaluation and follow-up were concluded by all patients. In comparison to the control group, the DES treatment group exhibited a significant increase in the rate of successful weaning (77.78% compared to 62.40%, *P* = .025, Table 2). In the DES treatment group, invasive ventilation and total ventilation times were shorter (9.8 ± 2.1 vs 11.2 ± 2.6 and 14.3 ± 2.7 vs 16.1 ± 3.4, respectively; both *P* < .01, Table 2). In the DES treatment group, there was a significant reduction in the occurrence of VAP (73.61% compared to 86.46%, *P* = .022, as shown in Table 2).

**Table 2:** Comparison of the primary aim.

	DES group (n = 72)	Control group (n = 133)	*P*
Weaning success rate	56 (77.78%)	83 (62.40%)	.025
Invasive ventilation time (d)	9.8 ± 2.1	11.2 ± 2.6	.001
Total time of ventilation (d)	14.3 ± 2.7	16.1 ± 3.4	.001
VAP incidence	53 (73.61%)	115 (86.46%)	.022

VAP = ventilator-associated pneumonia.

### 3.3. The secondary aim

**Figure 1.**
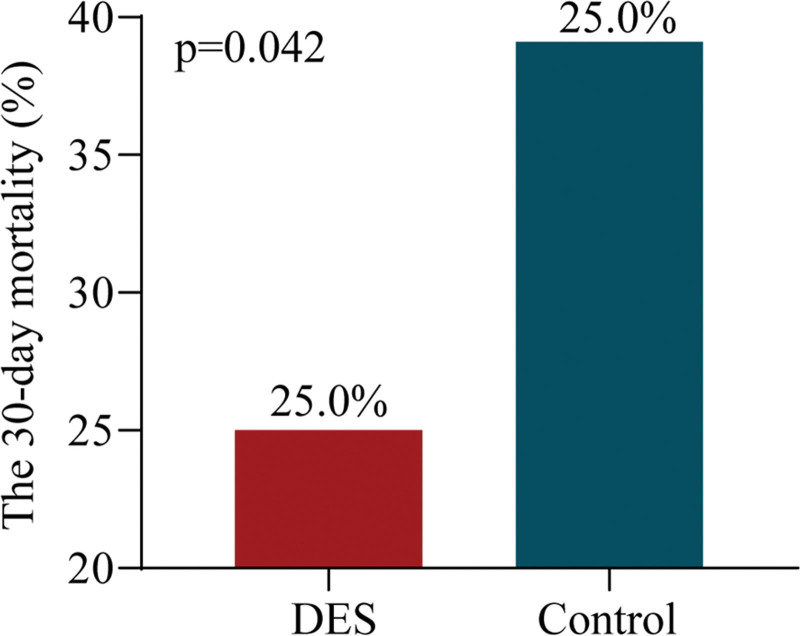
The mortality rate within a 30-d period for the 2 different groups.

**Figure 2.**
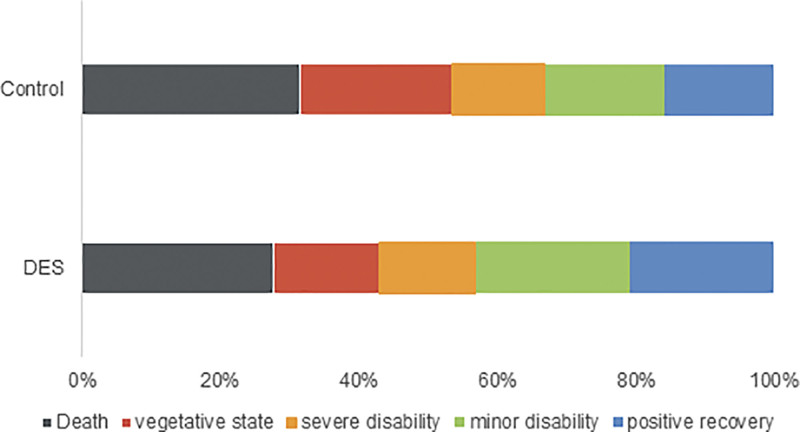
Follow-up of the outcome after a period of 6 mo.

In the DES treatment group, there was a significant decrease in 30-day mortality compared to the control group (*P* < .05, Figure 1). After 6 months of observation, we observed no notable disparity between the 2 groups in terms of the 6-month GOS score (*P* > .05, as depicted in Figure 2).

### 3.4. Safety evaluation

The most frequent adverse outcomes of DES were objective signs of tissue or neurovascular damage and discomfort. Following DES treatment, 6 individuals encountered intense discomfort, which was subsequently alleviated upon modifying the frequency of stimulation. Four patients encountered temporary tissue or neurovascular damage but self-repaired without requiring any intervention. Hence, DES treatment was safe and had no serious side effects.

### 3.5. Hospital stays and hospitalization costs

The DES group had a mean hospitalization period of 24.48 days, whereas the control group had a duration of 26.81 days. This difference was determined to be statistically significant (*P* = .001, Table 3). Furthermore, it was observed that the length of ICU stay decreased significantly in the DES group when compared to the control group (15.67 ± 3.76 vs 17.53 ± 4.28, *P* = .002, Table 3). The DES group had an average hospitalization cost of 11500 CNY (China Yuan), which was notably less than the control group expense of 13600 CNY (*P* = .001, Table 3).

**Table 3:** Comparison of Postoperative hospital stays and costs.

	DES (n = 72)	Control (n = 133)	*P*
Hospitalization stays, day, mean ± SD	24.48 ± 5.95	26.81 ± 6.39	.001
ICU stay, day, mean ± SD	15.67 ± 3.76	17.53 ± 4.28	.002
Hospitalization costs, CNY*10^4^, mean ± SD	11.50 ± 3.15	13.6 ± 3.72	.001

CNY = Chinese Yuan, SD = standard deviation.

## 4. Discussion

In this study, we discovered that DESs can substantially enhance the rate of successful weaning, reduce the duration of invasive ventilation and overall ventilation time, lower the occurrence of VAP, and enhance the 30-day survival rate. Despite the lack of improvement in neurological outcomes after 6 months, treatment with DES can reduce the duration of hospital stays, ICU stays, and overall hospitalization expenses. Additionally, DES treatment was safe and had no serious side effects.

Respiratory problems resulting from spontaneous intracerebral hemorrhage are highly prevalent, particularly in individuals with hemorrhage in the brain stem. Patients with intracerebral hemorrhage (ICH) who necessitate invasive mechanical ventilation (IMV) and tracheostomy undergo comprehensive therapy and encounter extended periods of hospitalization. In animal experiments, the rapid decrease in diaphragmatic contractility caused by mechanical ventilation was related to oxidative stress, diaphragmatic atrophy, and fiber damage, and the decrease in contractility was also proportional to the time of mechanical ventilation.^[17]^ Research has additionally discovered that the degeneration caused by lack of use occurs at a rate 8 times higher in the diaphragm than in other skeletal muscles. This disparity could be attributed to the unique characteristics of the diaphragm. Moreover, the diaphragm contracts consistently and rhythmically during both sleep and wakefulness, making it more susceptible to atrophy when immobilized.^[18]^ According to Albert,^[19]^ there were 5.2 million instances of acute stroke hospitalizations in the United States between 2008 and 2017. Of these, 9.4% were treated solely with IMV, while 1.4% underwent tracheostomy. Patients who received IMV alone or tracheostomy experienced longer hospital stays than those without IMV. Additionally, compared with patients without IMV, patients with IMV alone and tracheostomy had higher hospitalization costs and mortality. Therefore, reliance on ventilators or the need for prolonged mechanical ventilation can result in unfavorable outcomes and substantial escalation in both hospital duration and expenses. It was necessary to improve the patient respiratory function by trying various techniques and eventually increasing the weaning success rate at an early time.

DES, the pacing electrode was placed on the outer margin 1/3 of the lower end of the sternocleidomastoid muscle, and the auxiliary electrode was attached to the second intercostal space of the midclavicular line at the most superficial position of the phrenic nerve. By applying electrical stimulation to the phrenic nerve using a surface electrode, the phrenic nerve excitability was enhanced, ensuring consistent contraction of the diaphragm, expanding the diaphragm range of motion, and successfully preventing phrenic insufficiency. This approach effectively addresses weaning challenges caused by phrenic insufficiency in patients, leading to a reduced duration of mechanical ventilation. Masmoudi^[20]^ demonstrated that tracheotomy was performed on 3 adult sheep and phrenic nerve electrodes were implanted at both ends of the unilateral diaphragm muscle. After mechanical ventilation for 2 hours, diaphragm stimulation was initiated, and 30 minutes of diaphragm stimulation was given every 4 hours of mechanical ventilation. After 72 hours, the sheep were slaughtered, and samples were collected from both sides of the diaphragm for biopsy. The findings indicated a notable reduction in diaphragm damage and shrinkage on the stimulated side where the pacing electrode was applied. Furthermore, the muscle fibers of the diaphragm exhibited not only thickening but also an increase in fatigue-resistant fibers after pacing, leading to enhanced endurance and muscle strength. At present, animal experiments have confirmed the ameliorative effect of DESs on respiratory disorders and the help of early weaning mechanical ventilation.

DESs have the advantages of being noninvasive, simple, and passive and are easily accepted by patients. DESs can enhance respiratory function and alleviate hypoxia in individuals with pulmonary disorders, thereby enhancing athletic stamina.^[21]^ Observation of the DES impact on diaphragmatic motion was conducted on individuals without health issues, revealing a noteworthy enhancement in the extent of diaphragmatic movement after DES therapy. In normal individuals, DES can enhance the immediate displacement of the diaphragm by 1.3 cm, while in patients, it ranges from 0.48 to 1.22 cm. As a result of applying DES for 30 minutes every day for 20 consecutive days, the range of diaphragm motion increased by 0.34 to 1.12 cm.^[22]^ Michal Soták^[23]^ reported that stimulating the phrenic nerve to induce diaphragm contraction not only decreases the rate of muscle wasting during mechanical ventilation but also increases muscle thickness, which is the key to successfully weaning ventilator-dependent patients. In addition, Liu presented a potential randomized controlled trial indicating that patients on mechanical ventilation experience advantages from the combination of DES and early rehabilitation therapy in terms of reducing muscle atrophy and enhancing muscle strength.^[14]^ For prolonged mechanical ventilation patients, DES can significantly increase respiratory muscle strength and may be useful to facilitate weaning in this population.^[24]^ A systematic review discovered that DES was both safe and effective for patients with traumatic high cervical injuries and ventilator-dependent respiratory failure. Additionally, it has been suggested that earlier treatment could enhance effectiveness.^[25]^ Nonetheless, limited research has been conducted regarding ventilator reliance following ICH, and even fewer studies have explored the utilization of DESs in individuals experiencing respiratory failure subsequent to ICH. According to the current research, DES has been shown to enhance the rate of successful weaning, reduce the duration of invasive ventilation and overall ventilation time, and lower the occurrence of VAP.

The present study had certain constraints. The results of this study were based on a retrospective design conducted at a single center. Some patients’ files did not contain data on specific characteristics, leading to potential information bias. It is also important to consider the limitations of the monocentric approach. Furthermore, ultrasound did not assess the thickness and strength of the diaphragm in this investigation. It is necessary to design large sample multicenter randomized control trials to confirm these findings.

## 5. Conclusion

According to our study, DES has the potential to enhance the rate of successful weaning, reduce the duration of invasive ventilation and overall ventilation time, lower the occurrence of VAP, and enhance the 30-day survival rate. In addition to reducing hospital stays, ICU stays, and hospitalization costs, DES treatment can also have a positive impact on the duration of hospital stays, the length of stay in the ICU, and the overall expenses incurred during hospitalization. Nevertheless, our findings provide a basis for further investigation. Nevertheless, additional extensive multicenter randomized controlled trials are required to validate these discoveries.

## Author contributions

**Conceptualization:** Yan Wu, Yuhai Wang.

**Data curation:** Suqin Wang, Jing Zhang, Yan Wang.

**Formal analysis:** Yan Wu, Suqin Wang, Jing Zhang, Yuhai Wang.

**Investigation:** Yan Wu, Suqin Wang, Yan Wang, Jiaojiao Zhong, Yuhai Wang.

**Methodology:** Yan Wu, Suqin Wang, Jing Zhang, Yan Wang, Jiaojiao Zhong.

**Project administration:** Yuhai Wang.

**Resources:** Jing Zhang, Jiaojiao Zhong, Yuhai Wang.

**Software:** Suqin Wang, Yan Wang, Jiaojiao Zhong.

**Supervision:** Yan Wu, Suqin Wang, Jiaojiao Zhong, Yuhai Wang.

**Validation:** Yan Wu, Jing Zhang, Jiaojiao Zhong, Yuhai Wang.

**Visualization:** Yan Wu, Suqin Wang, Jing Zhang, Yan Wang, Yuhai Wang.

**Writing – original draft:** Yan Wu, Suqin Wang, Jing Zhang, Yan Wang, Jiaojiao Zhong, Yuhai Wang.

**Writing – review & editing:** Yan Wu, Yuhai Wang.
